# FOLFIRI-bevacizumab as a second-line treatment for advanced biliary tract cancer after gemcitabine-based chemotherapy

**DOI:** 10.3389/fonc.2023.1293670

**Published:** 2023-11-30

**Authors:** Nicolas Roussot, Julie Vincent, Remi Palmier, Guillaume Constantin, Leila Bengrine, Jean-David Fumet, François Ghiringhelli

**Affiliations:** ^1^ Department of Medical Oncology, Centre Georges-François Leclerc, Dijon, France; ^2^ Health Sciences Department, University of Burgundy, Dijon, France; ^3^ Cancer Biology Transfer Platform, Centre Georges-François Leclerc, Dijon, France; ^4^ Equipe TIRECs, Labellisée Ligue Contre le Cancer, Centre de Recherche INSERM LNC-UMR1231, Dijon, France; ^5^ Department of Biostatistics, Centre Georges-François Leclerc, Dijon, France; ^6^ Genetic and Immunology Medical Institute, Dijon, France

**Keywords:** FOLFIRI, FOLFIRI plus bevacizumab, bevacizumab, chemotherapy, second-line, advanced biliary tract cancer, metastatic biliary tract cancer, biliary tract cancer

## Abstract

**Background:**

Advanced biliary tract cancer (BTC) has a poor prognosis. Gemcitabine with platinum chemotherapy was the standard first-line chemotherapeutic regimen until the recent addition of anti-PD-1/PD-L1 antibodies. After disease progression, the only second-line chemotherapy that has demonstrated a survival benefit versus supportive care is FOLFOX (folinic acid, fluorouracil, and oxaliplatin), with a modest benefit. This study aimed to assess the efficacy and safety of second-line FOLFIRI (folinic acid, fluorouracil, and irinotecan) combined with bevacizumab for advanced BTC.

**Methods:**

This single-center retrospective study enrolled patients with metastatic BTC (intrahepatic cholangiocarcinoma [ICC], extrahepatic cholangiocarcinoma [ECC], or gallbladder carcinoma) that progressed after first-line gemcitabine-based chemotherapy. FOLFIRI-bevacizumab was administered intravenously every 2 weeks [folinic acid 200 mg/m², fluorouracil 400 mg/m² (bolus), fluorouracil 2400 mg/m² (46-h continuous intravenous infusion), irinotecan 180 mg/m², and bevacizumab 5 mg/kg] until unacceptable toxicity, patient refusal, or disease progression.

**Results:**

Overall, 28 patients received the FOLFIRI-bevacizumab regimen after gemcitabine-based chemotherapy. The median overall survival (OS) was 9.0 months (95% CI 6.4–16.5). The OS rate was 39.3% (95% CI 24.8–62.3) and 10.7% (95% CI 3.7–32.1) at 12- and 24-months respectively. The median progression-free survival (PFS) was 5.2 months (95% CI 3.1–10.2) with FOLFIRI-bevacizumab. The PFS rates at 12 months and 24 months were 17.9% (95% CI 8.19–39.5] and 10.7% (95% CI 3.7–31.2), respectively. The overall response rate (ORR) to FOLFIRI-bevacizumab was 23.1%, with a disease control rate (DCR) of 69.3%. Grade 3-4 adverse events (sAE) were reported in 20 patients (71.4%) treated with FOLFIRI-bevacizumab.

**Conclusion:**

FOLFIRI-bevacizumab as a second-line treatment for advanced BTC after gemcitabine-based chemotherapy showed efficacy and safety with a promising tumor response rate in this retrospective single-center study.

## 1 Introduction

Biliary tract cancer (BTC), which encompasses intrahepatic cholangiocarcinoma (ICC), extrahepatic cholangiocarcinoma (ECC), and gallbladder cancer, has been known to have a poor prognosis (1), with an increase in incidence in high-income countries. To date, surgery is the only curative therapy available for patients with resectable tumors. Liver resection for intrahepatic BTC provides a 5-year overall survival (OS) rate of 25–40% with a median OS of 40 months (2). Notably, most patients are diagnosed with unresectable locally advanced or metastatic BTC and are therefore no longer candidates for this therapeutic option (3). Thus, most patients receive systemic palliative care. Until recently, the first-line standard therapy for metastatic disease was based on a combination of platinum and gemcitabine (4), with cisplatin (5) or oxaliplatin (6–8). In randomized phase III studies, the combination of gemcitabine with cisplatin provided a median OS of 11.7 months (9), numerically better results than oxaliplatin with only 9.5 months of median OS (10). Immunotherapy adjunction with chemotherapy using anti-PD-L1 durvalumab prolongs the survival, with a median OS exceeding 1 year, irrespective of PD-L1 expression (11). Similar results were obtained with pembrolizumab (12). These chemoimmunotherapy protocols are now considered the standard first-line treatment for advanced BTC (13). After disease progression, the ABC-06 trial is the only randomized phase III study that has demonstrated an improvement in OS with FOLFOX versus supportive care (14). Nonetheless, the efficacy of FOLFOX in the second-line setting of metastatic BTC remains modest, with a median progression-free survival (PFS) and median OS of 4.0 and 6.2 months, respectively, and a poor objective response rate of 5%. Because the evidence for irinotecan-based therapies is currently limited, we report the efficacy and safety of the FOLFIRI-bevacizumab combination as a second-line treatment for advanced BTC after gemcitabine-based chemotherapy.

## 2 Materials and methods

### 2.1 Patients

This single-center retrospective study was conducted at the Georges-François Leclerc Center in Dijon, France and included patients with advanced BTC treated between 2009 and 2022. The patients were required to have a histologically confirmed diagnosis of BTC with metastatic or unresectable disease that progressed after first-line gemcitabine-based chemotherapy. Progression was confirmed using thoracic, abdominal, and pelvic computed-tomography (CT) scans. Only patients who received FOLFIRI-bevacizumab as a second-line treatment in a metastatic setting were included in the analyses. The off-label use of this treatment regimen was validated by a local multidisciplinary staff member.

### 2.2 Treatment regimen

Physical examination, complete blood cell count, and serum chemistry were performed before the initiation of chemotherapy. The FOLFIRI-bevacizumab regimen consisted of bevacizumab injection (5 mg/kg over 90 min) followed by irinotecan (180 mg/m² *i.v*. over 90 min) concurrently with folinic acid (200 mg/m² *i.v*. over 120 min), followed by fluorouracil (400 mg/m² *i.v*. bolus) and fluorouracil (2400 mg/m² IV infusion over 46 h). This treatment was administered every two weeks. Adverse events were graded according to the Common Terminology Criteria for Adverse Events version 5.0 (CTCAE 5.0). Dose reduction or treatment suspension was based on the grade of adverse events. The treatment was continued until unacceptable toxicity, patient refusal, or disease progression was observed.

### 2.3 Follow-up

Before every FOLFIRI-bevacizumab cycle, a complete physical examination, complete blood cell counts, and serum chemistry were performed. The tumor response was assessed every four cycles or when clinical progression was suspected according to symptoms and clinical examination with thoracic, abdominal, and pelvic CT-scans and determined using RECIST version 1.1. If a CT-scan does not provide a proper assessment of the tumor response, magnetic resonance imaging (MRI) can be performed.

### 2.4 Statistical analyses

Progression-free survival (PFS) at the second-line was calculated from the date of FOLFIRI-bevacizumab initiation to the date of progression or death. The overall survival (OS) at second-line treatment was calculated from the date of FOLFIRI-bevacizumab initiation to the date of death from any cause or censored at the date of the last follow-up. PFS at first-line treatment was calculated from the date of diagnosis of advanced biliary tract cancer to the date of progression or death, and OS at first-line treatment was calculated from the date of diagnosis to the date of death from any cause or censored at the date of last follow-up. The curves were plotted using a Kaplan–Meier analysis.

## 3 Results

### 3.1 Patient characteristics

Overall, 28 patients received FOLFIRI-bevacizumab as a second-line treatment for advanced BTC. A combination of gemcitabine and oxaliplatin (n=20, 71.4%) was the most frequently used first-line regimen (**Table 1**). The median age was 69.0 years and the majority of patients were male (n=16; 57.1%). Most patients had intrahepatic disease (n=19; 67.9%), with a similar proportion of extrahepatic disease (n=4; 14.3%) and gallbladder disease (n=5; 17.9%). Among the enrolled patients, 16 (57.1%) patients were diagnosed with metastatic disease, 8 (28.6%) patients with unresectable locally advanced disease, and 4 (14.3%) patients with resectable BTC. One patient underwent gallbladder removal for cholecystitis, which turned out to be an adenocarcinoma with peritoneal carcinomatosis. The mean baseline CA19-9 level was 1387.3 kU/L. Notably, 16 (57.1%) patients underwent molecular testing for advanced BTC. The genomic characteristics of these patients are shown in **Figure 1**.

**Table 1:** Baseline characteristics.

	FOLFIRI-BEVACIZUMAB 2nd line (n=28)
Sex	
Male	16 (57.1%)
Female	12 (42.9%)
Age	
Median [min - max]	69.0 [45.0 - 81.0]
Disease stage at diagnosis	
Localized resectable	4 (14.3%)
Locally advanced unresectable	8 (28.6%)
Metastatic	16 (57.1%)
Tumor site	
Intrahepatic	19 (67.9%)
Gallbladder	5 (17.9%)
Extrahepatic	4 (14.3%)
Tumor site among extrahepatic disease	
Distal	1 (20.0%)
Peri-hilar	4 (80.0%)
Histology	
Adenocarcinoma	27 (96.4%)
Adenosquamous	1 (3.6%)
Grade of differentiation	
Well	4 (21.1%)
Moderately	10 (52.6%)
Poorly	5 (26.3%)
Not specified	9
ECOG performance status	
0	9 (37.5%)
1	13 (54.2%)
2	2 (8.3%)
Missing	4
**Underwent previous surgery of the primary tumor**	5 (17.9%)*
**Underwent previous transarterial chemoembolization (TACE)**	5 (17.9%)
**Underwent previous transarterial radioembolization (TARE)**	2 (7.1%)
**Underwent previous adjuvant chemotherapy**	3 (10.7%)
Capecitabine Gemcitabine Gemcitabine-oxaliplatin	1 (33.3%) 1 (33.3%) 1 (33.3%)
Previous 1st line systemic chemotherapy	
Gemcitabine-oxaliplatin	20 (71.4%)
Gemcitabine-cisplatin	5 (17.8%)
Gemcitabine-carboplatin	1 (3.6%)
Gemcitabine	1 (3.6%)
FOLFIRINOX	1 (3.6%)#
**Intraperitoneal chemotherapy**	1 (3.6%)
Baseline CA19.9 (kU/L)	
Mean (std)	1387.3 (4731.3)
Median [min - max]	76.8 [4.0 - 19670.0]
Missing	11
Neutrophils/lymphocytes ratio (NLR)	
Mean (std)	5.0 (2.5)
Median [min - max]	4.7 [1.4 - 9.1]
Missing	10

*5 patients received surgery for resectable BTC. One patient underwent gallbladder removal for cholecystitis suspicion, which turned out to be an adenocarcinoma with peritoneal carcinomatosis.

#1 patient was treated with FOLFIRINOX in the clinical trial PRODIGE 38 AMEBICA which randomized modified FOLFIRINOX versus gemcitabine-cisplatin.

**Figure 1:**
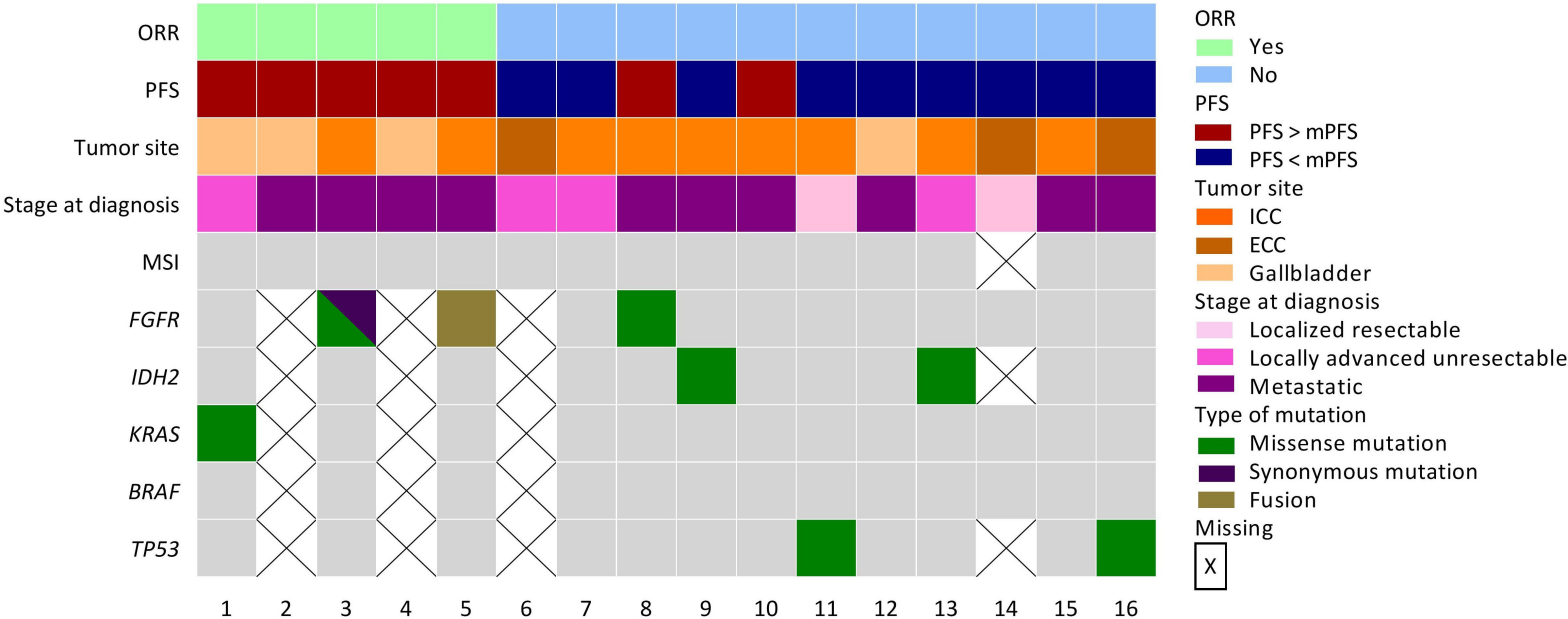
Genomic characterization of patients. Oncoplot representing genomic landscape among patients that have received molecular testing (n=16; 57.1%). * Patient 3 was diagnosed with a *FGFR1* missense mutation (c.2223C>A; p.Phe741Leu) and with a *FGFR3* synonymous mutation (c.933G>A; p.Thr311Thr).

### 3.2 Efficacy of the first-line treatment

In the first-line setting of metastatic disease, 26 (92.8%) patients were treated with a combination of gemcitabine and a platinum derivative (oxaliplatin for 20 (71.4%) patients, cisplatin for five (17.8%) patients, and carboplatin (3.6%) for one patient) (**Table 1**). The median OS from the first line of systemic chemotherapy was 16.5 months (95% CI 12.3–25.7). The OS rate was 67.9% (95% CI 52.6–87.6) at 12-months and 32.1% (95% CI 18.8–55.1) at 24-months (**Supplemental Figure 1A**). The median PFS was 7.6 months (95% CI 3.4–9.5). The PFS rates at 12 months and 24 months were 25.0% (95% CI 13.2–47.5) and 10.7% (95% CI 3.7–31.2), respectively (**Supplemental Figure 1B**). Among the 27 (96.4%) patients assessable for tumor response, the ORR was 42.9%, and the disease control rate (DCR) was 67.9% (**Supplemental Table 1**). The median OS tended to be longer for gallbladder cancer with 28.6 months (95% CI 21.7-not evaluable [NE]) than for ICC with 16.4 months (95% CI 10.3–36.9) and ECC with 12.9 months (95% CI 12.3–NE) (log-rank *p*-value = 0.1944, **Supplemental Figure 2A**). The median PFS for ICC, ECC, and gallbladder cancer were 5.6 (95% CI 2.5–14.3), 8.6 (95% CI 5.0–NE), and 10.1 months (95% CI 2.6–NE) (log-rank *p*-value = 0.7766, **Supplemental Figure 2B**), respectively. Patients who received local treatment for BTC, that is, previous surgery of the primary tumor, transarterial chemoembolization (TACE), or transarterial radioembolization (TARE), tended to have a longer OS than those who did not, although the PFS was similar (**Supplemental Figures 3A, B**).

### 3.3 Efficacy of FOLFIRI-bevacizumab in the second line setting

**Figure 2:**
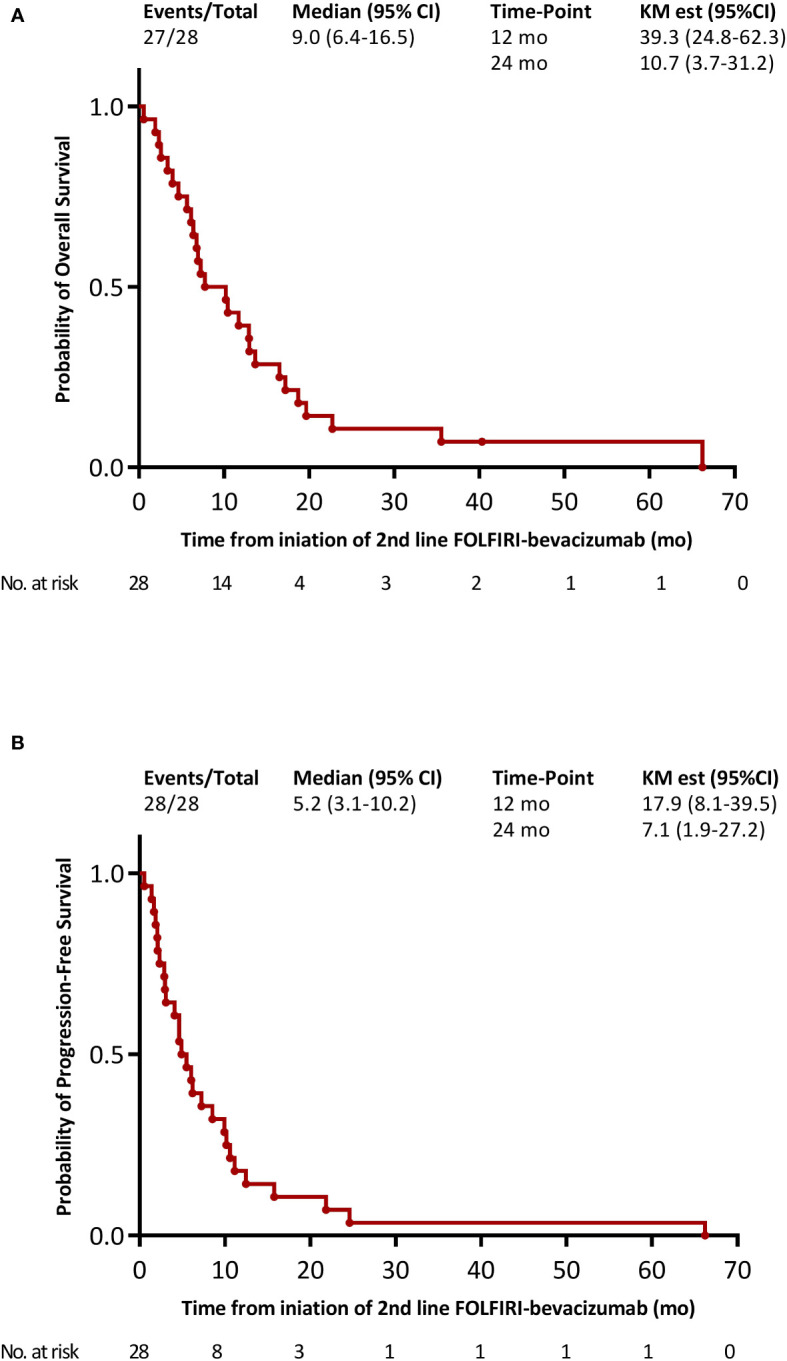
Kaplan-Meier survival curves with FOLFIRI-bevacizumab as the 2nd line treatment for metastatic disease. **(A)** overall survival; **(B)** progression-free survival.

**Figure 3:**
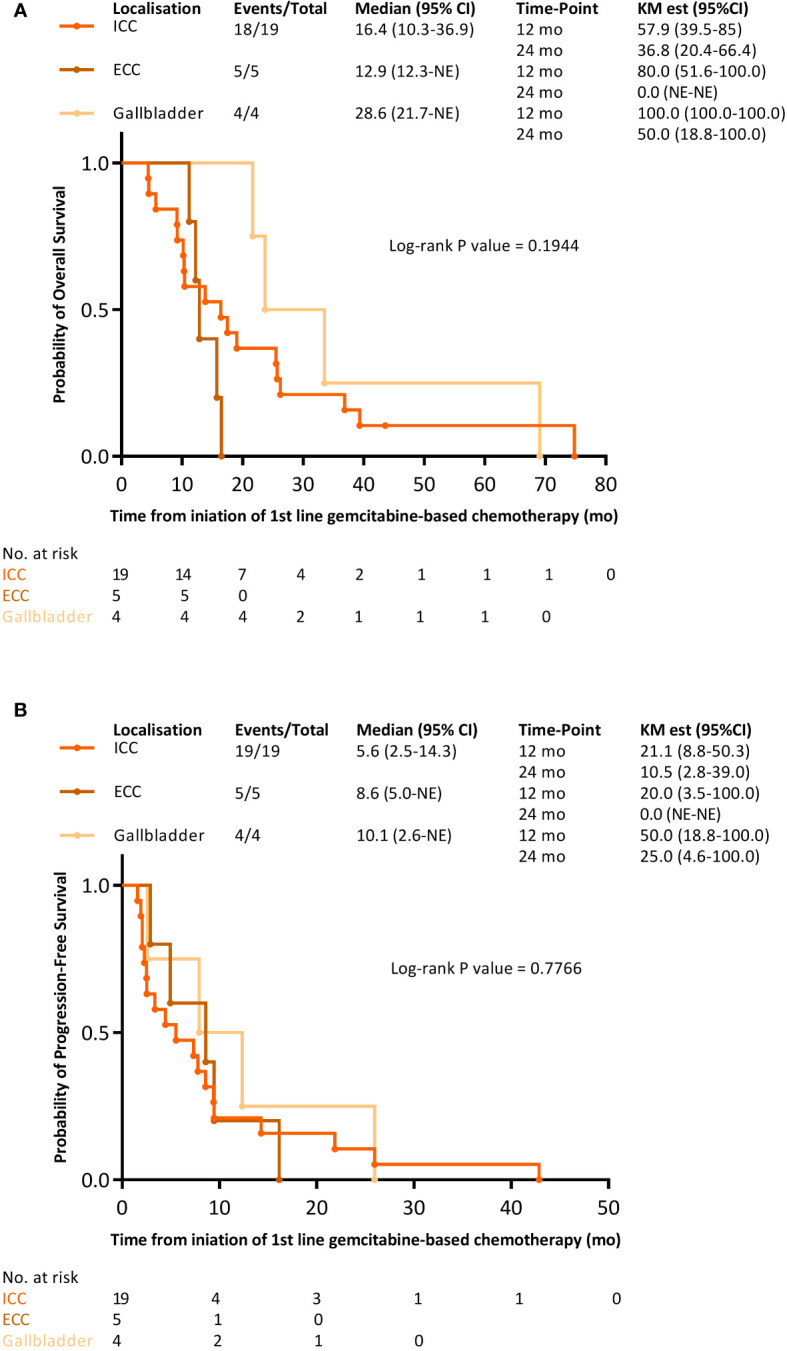
Kaplan-Meier survival curves with FOLFIRI-bevacizumab as the 2^nd^ line treatment for metastatic disease within distinct tumor sites. **(A)** overall survival; **(B)** progression-free survival.

**Figure 4:**
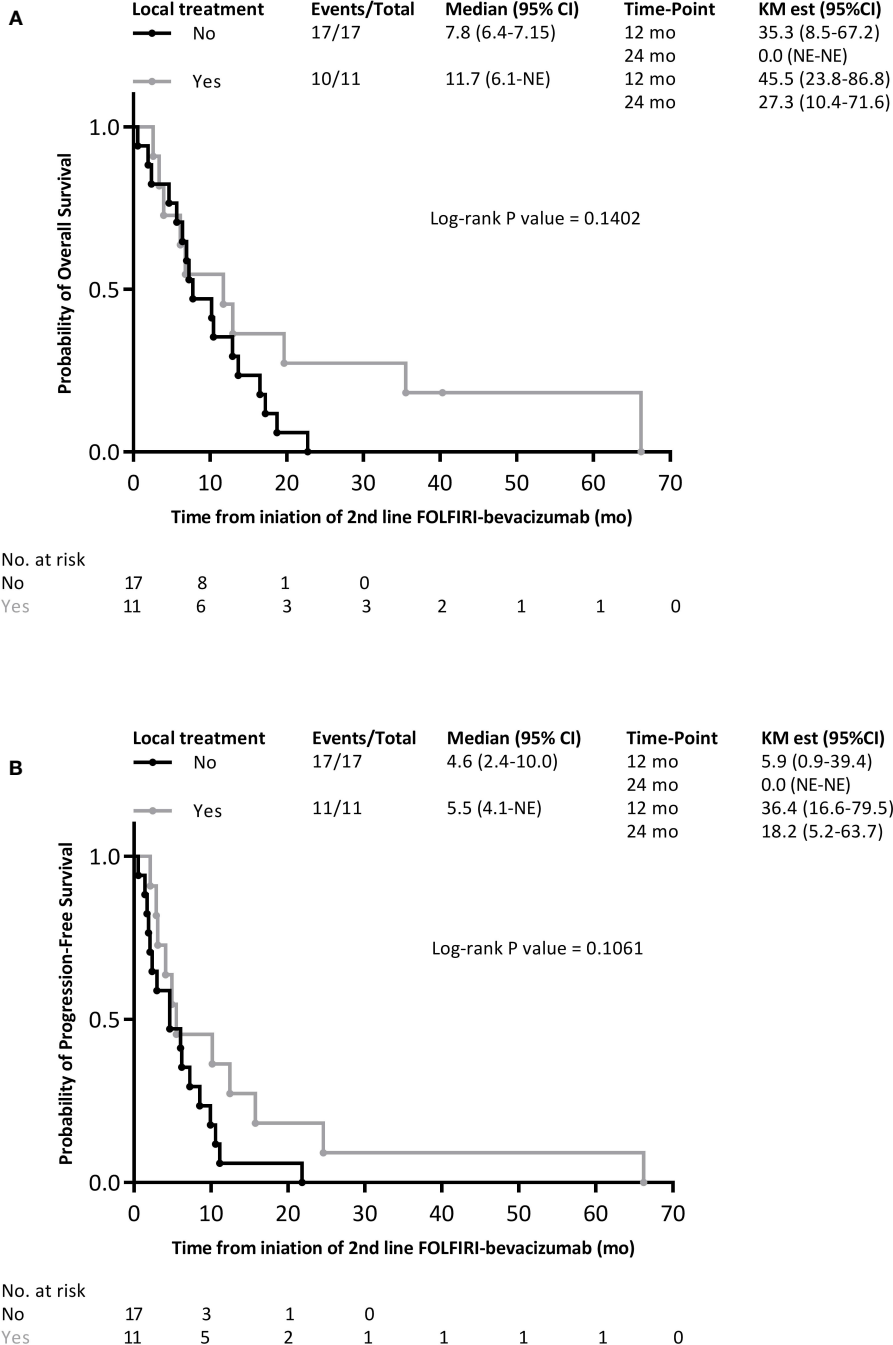
Kaplan-Meier survival curves with FOLFIRI-bevacizumab as the 2^nd^ line treatment for metastatic disease within local treatment subgroups/. **(A)** overall survival; **(B)** progression-free survival.

The median OS from the initiation of FOLFIRI-bevacizumab as a second-line treatment for metastatic disease was 9.0 months (95% CI 6.4–16.5). The OS rate was 39.3% (95% CI 24.8–62.3) at 12-months and 10.7% (95% CI 3.7–32.1) at 24-months (**Figure 2A**). The median PFS was 5.2 months (95% CI 3.1–10.2) with FOLFIRI-bevacizumab. The PFS rates at 12 months and 24 months were 17.9% (95% CI 8.1–39.5) and 10.7% (95% CI 3.7–31.2), respectively (**Figure 2B**). Of the 28 patients treated with FOLFIRI–bevacizumab, 26 (92.3%) patients were assessed for tumor response (**Table 2**). The ORR with FOLFIRI-bevacizumab was 21.4%, with six patients achieving a partial response. However, complete responses were not observed. A total of 42.9% (n=12) of the patients had stable disease with FOLFIRI-bevacizumab, which led to a DCR of 64.3%. The median OS was significantly different within distinct tumor sites (log-rank *p* = 0.0373, **Figure 3A**). Patients with metastatic gallbladder cancer had the longest median OS of 15.9 months (95% CI 10.5–NE). Patients with metastatic ICC had 10.2 months (95% CI 6.8–19.7) of median OS. Finally, metastatic ECC was the tumor site with the worst prognosis, with a median OS of 6.1 months (5.7–NE). The median PFS tended to be longer for gallbladder cancer than for ICC and ECC, at 11.8 (95% CI 3.0–NE), 4.9 (95% CI 2.9–10.6) and 4.6 months (95% CI 3.1–NE) (log-rank *p*-value = 0.0956, **Figure 3B**). Patients that have received local treatment for BTC tended to have a longer OS than those who did not, with 11.7 (6.1–NE) and 7.8 months (6.4–7.15), respectively (logrank *p*-value = 0.1402, **Figure 4A**). A similar trend was observed for PFS (log-rank test, *p*-value = 0.1061; **Figure 4B**). Two patients diagnosed with *TP53* missense mutations had a particularly short PFS of 1.68 and 4.93 months.

**Table 2:** Overall response rate with FOLFIRI-bevacizumab as the 2^nd^ line treatment for metastatic disease.

Tumor response with 2^nd^ line FOLFIRI-BEVACIZUMAB	N (%)
Partial response	6 (21.4%)
Stable disease	12 (42.9%)
Progressive disease	6 (28.6%)
Non evaluable	2 (7.1%)

### 3.4 Safety

Anemia (n=19, 67.9%), nausea (n=14, 50%), bleeding (n=14, 50%), and diarrhea (n=14, 50%) were the most frequent mild AEs. Grade 3 or higher adverse events (sAEs) occurred in 20 (71.4%) patients who received FOLFIRI-bevacizumab as the second-line treatment for metastatic disease. The most frequent hematological and non-hematological severe AEs were neutropenia (n=8, 28.6%) and infection (n=13, 46.4%), respectively. Only 2 (7.1%) grade ≥ 3 bleedings occurred. One patient had a gastric/duodenal hemorrhage related to gastric and duodenal ulcers that required transfusion. The other case involved grade 4 digestive bleeding related to esophageal varices that required transfusion and endoscopic ligature. Notably, 4 (14.3%) grade ≥ 3 hypertension occurred with FOLFIRI-bevacizumab but no grade ≥ 3 proteinuria. No grade 5 adverse events were reported. **Table 3** summarizes the frequencies of AEs.

**Table 3:** Safety with FOLFIRI-bevacizumab.

	NCI-CTC grade	
	All grades	Grade ≥ 3
	(n=28)	
**Hematological**		
Anemia	19 (67.9%)	3 (10.7%)
Neutropenia	11 (39.3%)	8 (28.6%)
Thrombocytopenia	18 (64.3%)	0 (0%)
**Non hematological**		
Nausea	14 (50.0%)	1 (3.6%)
Mucitis	5 (17.9%)	1 (3.6%)
Diarrhea	14 (50.0%)	2 (7.1%)
Infection	6 (21.4%)	13 (46.4%)
Hypertension	9 (32.1%)	4 (14.3%)
Proteinuria	8 (28.6%)	0 (0%)
Bleeding	14 (50.0%)	2 (7.1%)

## 4 Discussion

The results of this single-center retrospective study suggest that the combination of FOLFIRI with bevacizumab is an active and safe second-line regimen in patients with advanced BTC who show disease progression with gemcitabine-based chemotherapy.

In this study, the majority of patients received a combination of gemcitabine and oxaliplatin as the first-line systemic treatment for metastatic disease. The first-line standard of care for advanced BTC consists of a gemcitabine-based combination therapy (15), with cisplatin being the preferred option (5). Oxaliplatin remains an alternative to cisplatin when renal function is a concern (13). Nevertheless, the median PFS in the first-line setting observed in this cohort was slightly longer than that previously published in studies reporting the efficacy of the gemcitabine-oxaliplatin combination (10, 16, 17) and close to the 8.0 months reached with gemcitabine and cisplatin (9). Before immunotherapy adjunction demonstrated improvement in survival in the first-line setting in the TOPAZ- (11) and Keynote-966 (12) phase III studies, neither targeted therapy addition (10, 18) nor chemotherapy intensification with modified FOLFIRINOX (19) or the addition of albumin-bound paclitaxel succeeded in improving survival. In Asia, the addition of S-1 to gemcitabine and cisplatin in the first-line setting prolongs survival compared to the chemotherapy doublet (20). The IMBrave-151 (21) phase II trial assessed the combination of atezolizumab, bevacizumab, gemcitabine, and cisplatin and showed promising preliminary results; however, it lacked an appropriate control group with gemcitabine and cisplatin.

From a molecular perspective, BTC is a heterogeneous disease with distinct genomic and epigenomic landscapes (22, 23). Based on this molecular dismemberment, targeted therapies have been used to improve outcomes beyond first-line therapy. In the present study, more than half of the patients underwent molecular testing. Two of these patients were diagnosed with FGFR pathway alterations. As expected in patients with such features, the type of BTC was ICC (24). One patient was observed to have a FGFR1 missense mutation (c.2223C>A; p.Phe741Leu) along with a synonymous FGFR3 mutation (c.933G>A; p.Thr311Thr), while the second patient was diagnosed with a FGFR2 fusion (FGFR2-C5orf15) that would have rendered him a candidate for targeted therapies since pemigatinib (25), infigratinib (26), or futibatinib (27, 28) are validated options for patients with FGFR2 fusions (13). One patient harbored a IDH2 mutation; however, ivosidenib (29, 30) has only been proposed for patients with IDH1 mutations. Patients harboring BRAF mutations are candidates for dabrafenib with trametinib (31). The amplification of HER2/neu is targeted by trastuzumab*, which is* either associated with pertuzumab (32) or FOLFOX (33). Pembrolizumab is the preferred option for patients with mismatch repair deficiency (dMMR) or microsatellite instability (MSI-H) (34). Recently, adagrasib showed a promising efficacy in advanced BTC harboring a KRAS^G12C^ mutation, with an ORR of 41.7% (35). Globally, molecular screening techniques enable treatment with molecular targeted agents, which prolong survival in patients with advanced BTC (36).

The choice of second-line chemotherapy for patients without targetable genomic alterations was based on retrospective and non-randomized studies (37–39) until the ABC-06 trial established FOLFOX as the standard of care (13). After progression to first-line gemcitabine-based chemotherapy, nearly half of the patients are offered second-line chemotherapy. In a retrospective German study, nearly one-third of patients received FOLFOX/CAPOX (capecitabine-oxaliplatin) and less than a tenth was offered FOLFIRI or FOLFIRINOX (40). Studies that assessed the FOLFIRI regimen in this setting are summarized in **Table 4**. FOLFIRI-bevacizumab in this study allows reaching a median PFS and OS of 5.2 and 9.0 months, respectively, with a promising ORR of 21.4% and a DCR of 64.3%. Our study seems to be numerically comparable favorably with previous reports on therapy consisting of FOLFIRI alone. In the second-line setting, the ABC-06 (14) study was the only randomized phase III trial that demonstrated the survival benefit of FOLFOX over supportive care. Despite its modest efficacy, with a median PFS of 4.0 months, median OS of 6.2 months, and poor ORR of 5%, this regimen is the standard of care after gemcitabine-based chemotherapy for patients without actionable mutations (13). Irinotecan-based therapies using nanoliposomal irinotecan (Nal-iri) were assessed. NIFTY (50), a Korean phase IIb study, and NALIRICC (51), a German phase II study, are two clinical trials that compared Nal-iri with 5-FU to 5-FU alone after gemcitabine-based therapies. While the first met its primary endpoint with an improvement of the median PFS with the Nal-iri – 5-FU combination over 5-FU alone (7.1 months vs. 1.4 months; hazard ratio 0.56, 95% CI 0.39–0.81; *p*=0.0019), the second did not since the two regimens reached a median PFS of 2.76 and 2.3 months, respectively. These contradictory results highlight that biliary tract cancer presents with different biological features in Western and Asian countries, which may affect the response to therapy (52, 53). However, the ORR were similar for NIFTY and NALIRICC (14.8% and 14.3%, respectively). **Table 5** summarizes prospective randomized studies on second-line therapies for advanced BTC.

**Table 4:** non randomized studies that have assessed FOLFIRI in the second-line setting of advanced BTC.

Study	Sebbagh et al. (41)	Guion-Dusserre et al. (42)	Caparica et al. (43)	Mizrahi et al. (44)	Möhring et al. (45)	Chiaravalli et al. (46)	Huang et al. (47)	Müller et al. (48)	Balarine et al. (49)
Year	2015	2015	2019	2020	2021	2022	2022	2023	2023
Design	Single center retrospective	Single center retrospective	Single center retrospective	Multicenter retrospective	Single center retrospective	Single center retrospective	Single center	Single center retrospective	Single center retrospective
N	52	13	12	98	21 in 2^nd^ line	51	9	47 in 2^nd^ line	67 in 2^nd^ line
Treatment	FOLFIRI	FOLFIRI-bevacizumab	FOLFIRI	FOLFIRI (N=77, 79%), FOLFIRI-bevacizumab (N=13, 13%)	FOLFIRI (N=12, 57.1%), capecitabine/FOLFOX (N=5, 23.8%), (gemcitabine-cetuximab (N=4, 19%	FOLFIRI (N=28, 55%), FOLFOX (N=15, 29%), capecitabine (N=2 ; 4%), experimental drugs (N=6, 12%)	FOLFIRI HAIC (hepatic arterial infusion chemotherapy)	FOLFIRI (N=19, 40.4%), FOLFIRI+MIT (Minimal Invasive Therapies) (N=14, 28.8%)	FOLFIRI (N=25, 29.9%), FOLFOX (N=26, 38.8%)
Population	2^nd^ line treatment for mBTC after progression gemcitaine-oxaliplatin therapy	2^nd^ line treatment for mBTC after progression gemcitaine-oxaliplatin therapy	2^nd^ and further lines treatment for mBTC after progression on 1st line gemcitabine-platinum therapy	1^st^ and further lines treatment for mBTC (51% in 2^nd^ line setting)	2^nd^ line treatment for mBTC after progression on 1st line gemcitabine-platinum therapy	2^nd^ line treatment for mBTC after progression on 1st line gemcitabine-cisplatin therapy	2^nd^ and further lines treatment for unresectable BTC (ICC) after progression on 1st line gemcitabine-platinum-aPD-1 +/- lenvatinib regimen	2^nd^ line treatment for mBTC after progression on 1st line gemcitabine-cisplatin therapy	2^nd^ line treatment for mBTC after progression on 1st line gemcitabine-cisplatin therapy
Study	Sebbagh et al. (41)	Guion-Dusserre et al. (42)	Caparica et al. (43)	Mizrahi et al. (44)	Möhring et al. (45)	Chiaravalli et al. (46)	Huang et al. (47)	Müller et al. (48)	Balarine et al. (49)
mPFS	3.2 (95% CI 2.2–4.0)	8 mo (95%CI: 7-16)	1.7 months (95% CI; 0.66-2.67)	2.4 (95%CI: 1.8-3.7) in 2^nd^ line	N/A	3.5 mo (whole cohort)	5.0 mo (95% CI: 2.65–7.34)	2.79 mo (95% CI 2.5-4.2) with FOLFIRI vs 3.45 mo (3.028-3.872) with FOLFIRI-MIT (HR 1.057, 95% CI: 0.501-2.230, p= n.s.)	N/A
mOS	8.4 (95% CI 6.0–17.7)	20 mo (95%CI: 8-48).	5 months (95% CI; 2.77-7.20)	7.7 (95%CI: 4.9-10.5) in 2^nd^ line	7.1 months (95% CI: 0.35, 1.16) (whole cohort)	8.8 mo (whole cohort); mOS: 11.3 mo with FOLFIRI vs 5.4 mo with FOLFOX (HR 0.46, 95% CI: 0.18-0.88, p = 0.019)	8.0 mo (95% CI 6.04–9.96	3.35 (95% CI 2.802-6.458) with FOLFIRI vs 10.35 mo (3.284-17.416) with FOLFIRI-MIT (HR 0.411, 95% CI: 0.168-1.003, p= 0.021)	mOS: 8 mo (95% CI: 3.31 - 12.68) with FOLFIRI vs 5 mo (95% CI 0.68-9.32) with FOLFOX (p= 0.259)
ORR	N/A	38.4% (95%CI: 12.5-89	N/A	9.8% (whole cohort)	0% (whole cohort)	4% (whole cohort)	22.2%	N/A	N/A
DCR	N/A	84.5% (95%CI: 42-100)	17%	45.1% (whole cohort)	19% (whole cohort)	39% (whole cohort)	55.5%	N/A	N/A
≥ Grade 3 AE	N/A	30.7%	33.3%	Not provided	≥ 38.1% (whole cohort)	N/A	≥ 22.2%	19.4% with FOLFIRI vs 20.4% with FOLFIRI-MIT	56% with FOLFIRI vs 61.5% with FOLFOX (p=0.688)

NA, Not applicable.

**Table 5:** Prospective randomized studies for second-line treatment of advanced BTC.

Study	ABC-06 (14)	Choi et al (54)	NIFTY (50)	NALIRICC (51)
Year	2021	2021	2022	2022
Design	III	II	II	II
N	162	118	174	100
Treatment	FOLFOX vs ASC	mFOLFOX vs mFOLFIRI	Nal-iri + 5-FU vs 5-FU	Nal-iri + 5-FU vs 5-FU
Population	mBTC and progression on 1st line gemcitabine plus cisplatin therapy	mBTC and progression on 1st line gemcitabine plus cisplatin therapy	mBTC and progression on 1st line gemcitabine plus cisplatin therapy	mBTC and progression on 1st line gemcitabine-based therapy
1^st^ endpoint	OS	OS	BICR-assessed PFS	PFS
Results	Met	Unmet	Met	Unmet
	mOS: 6.2 mo with FOLFOX vs 5.3 mo with ASC	mOS: 6.3 mo with mFOLFOX and 5.7 mo with mFOLFIRI	BICR-assessed mPFS: 7.1 mo with Nal-iri + 5-FU/LV vs 1.4 mo with 5-FU/LV	mPFS: 2.76 mo with Nal-iri + 5-FU vs 2.3 mo with 5-FU/LV
HR (95% CI)	0·69 (0·50–0·97)	1.1 (0.7-1.6)	0·56 (0·39–0·81)	Not provided
ORR	5%	5.9% vs 4.0%	14.8% vs 5.8%	14.3% vs 3.9%
DCR	33%	66.7% vs 64%	64.8% vs 34.9%	Not provided
≥ Grade 3 AE	69%	55.4% with mFOLFOX vs 50.0% with mFOLFIRI	42% with Nal-iri + 5-FU vs 24% with 5-FU	70.8% with Nal-iri + 5-FU vs 50% 5-FU

In conclusion, our study on the FOLFIRI-bevacizumab regimen after progression to first-line gemcitabine-based therapy showed promising activity with a safety profile. The survival outcomes with this combination were similar to those achieved with FOLFOX, FOLFIRI, and Nal-IRI + 5FU. However, the adjunction of an anti-angiogenic agent with bevacizumab may provide a higher response rate. Hence, this regimen could benefit symptomatic patients with a massive tumor burden for whom obtaining a rapid response is crucial.

## Data availability statement

The raw data supporting the conclusions of this article will be made available by the authors, without undue reservation.

## Ethics statement

The studies involving humans were approved by loi Informatique et libertés de 1978 modifiée et le Règlement (UE) n°2016/679 relatif à la protection des données personnelles (RGPD). The studies were conducted in accordance with the local legislation and institutional requirements. The participants provided their written informed consent to participate in this study.

## Author contributions

NR: Conceptualization, Data curation, Investigation, Writing – original draft, Writing – review & editing. JV: Investigation, Writing – review & editing. RP: Investigation, Writing – review & editing. GC: Writing – review & editing, Formal Analysis, Software. LB: Investigation, Writing – review & editing. JF: Investigation, Writing – review & editing. FG: Conceptualization, Investigation, Supervision, Writing – review & editing.
