# Supplementary material for: FOLFIRI-bevacizumab as a second-line treatment for advanced biliary tract cancer after gemcitabine-based chemotherapy
Source: Front Oncol. 2023 Nov 30;13:1293670. doi: 10.3389/fonc.2023.1293670 (PMC10720590; doi:10.3389/fonc.2023.1293670)
Supplement: Supplementary file 1 [file Table_1.docx]

| **Tumor response with 1^st^ line gemcitabine-based chemotherapy** | **N (%)** |
| --- | --- |
| Partial response | 12 (42.9%) |
| Stable disease | 7 (25.0%) |
| Progressive disease | 8 (28.6%) |
| Non evaluable | 1 (3.6%) |

**Supplemental Table 1:** overall response rate with 1^st^ line gemcitabine-based chemotherapy for metastatic disease
