# Supplementary figures and images for: FOLFIRI-bevacizumab as a second-line treatment for advanced biliary tract cancer after gemcitabine-based chemotherapy
Source: Front Oncol. 2023 Nov 30;13:1293670. doi: 10.3389/fonc.2023.1293670 (PMC10720590; doi:10.3389/fonc.2023.1293670)

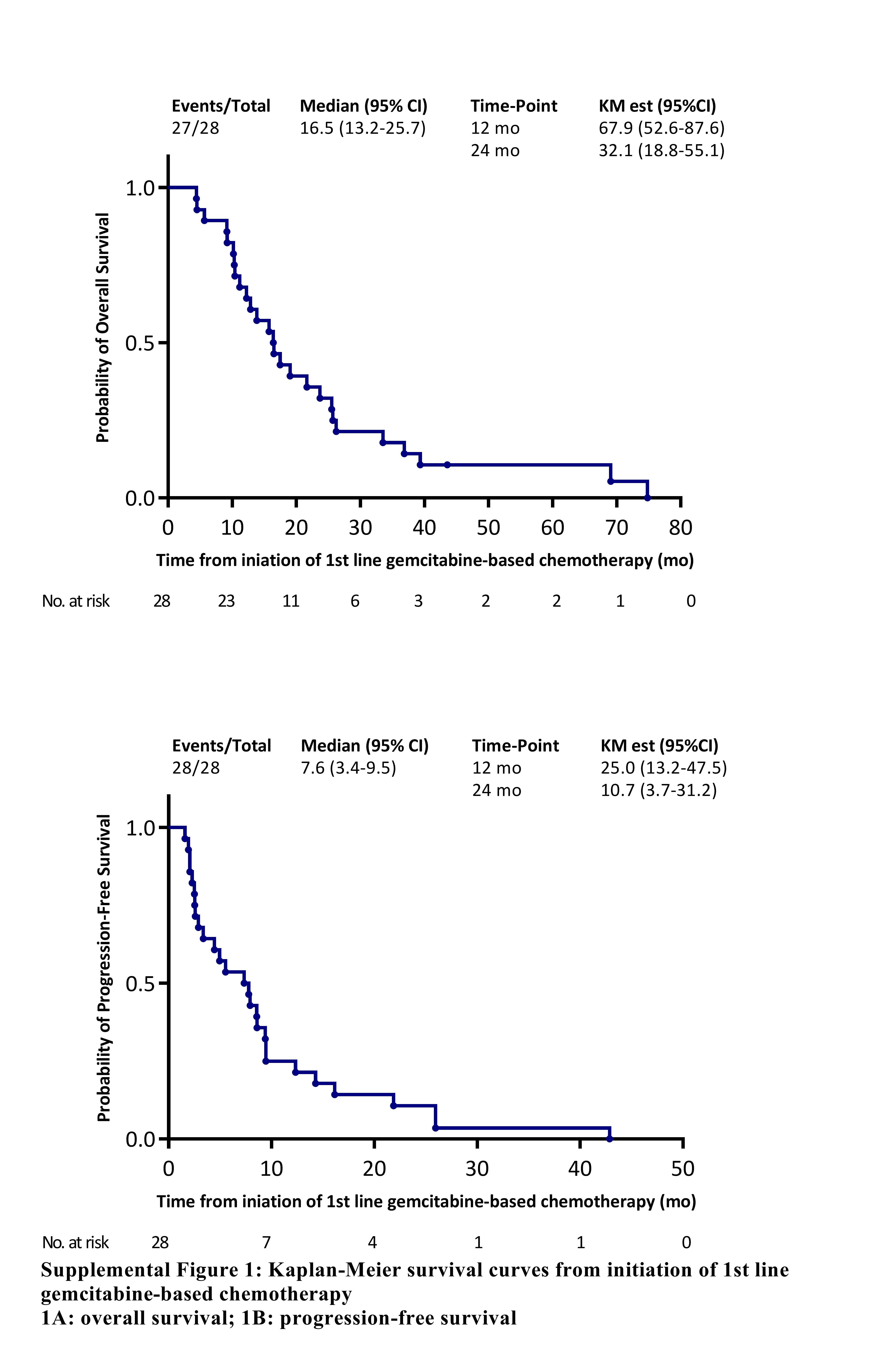

Supplement: Supplementary file 2 [file Image_1.jpeg]

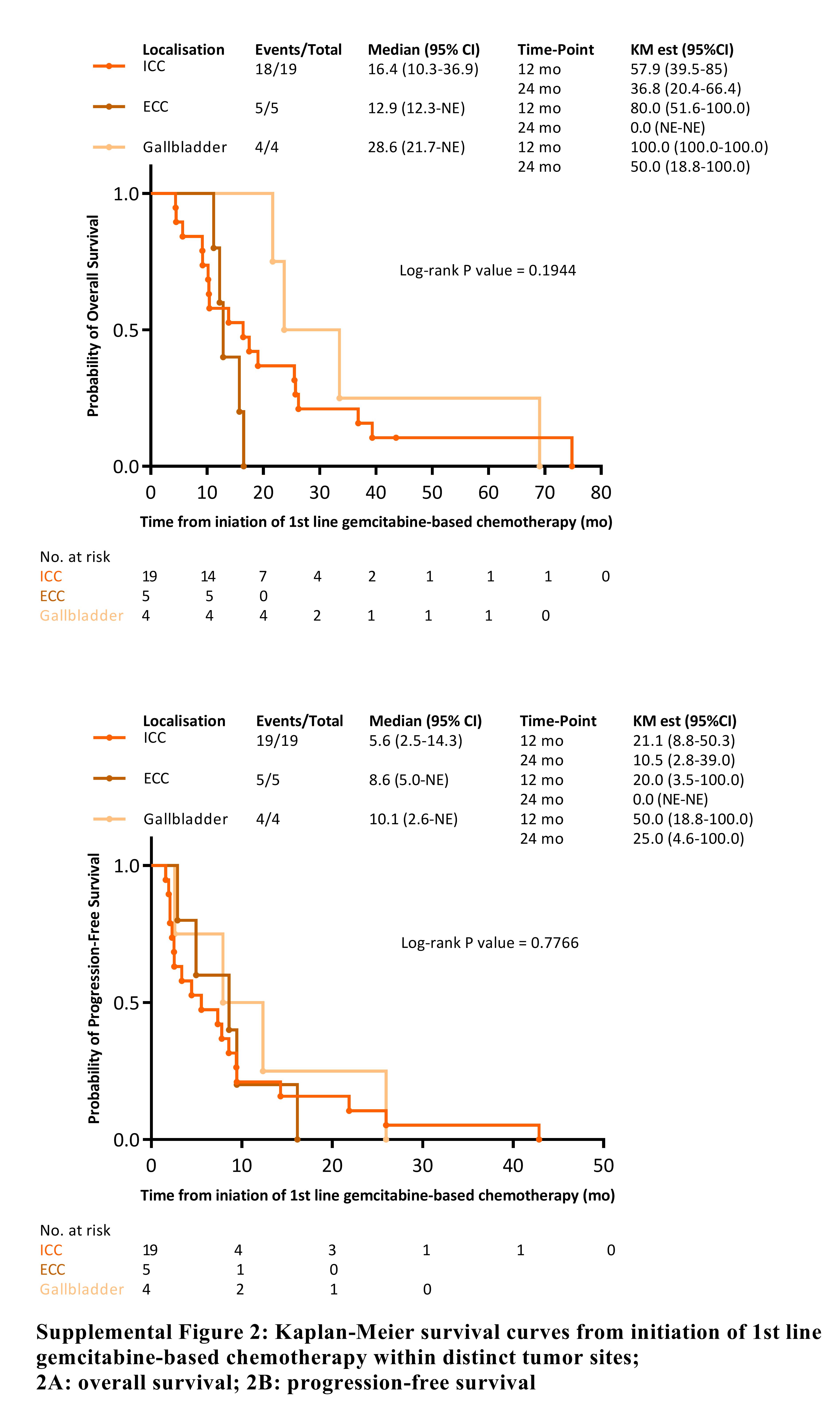

Supplement: Supplementary file 3 [file Image_2.jpeg]

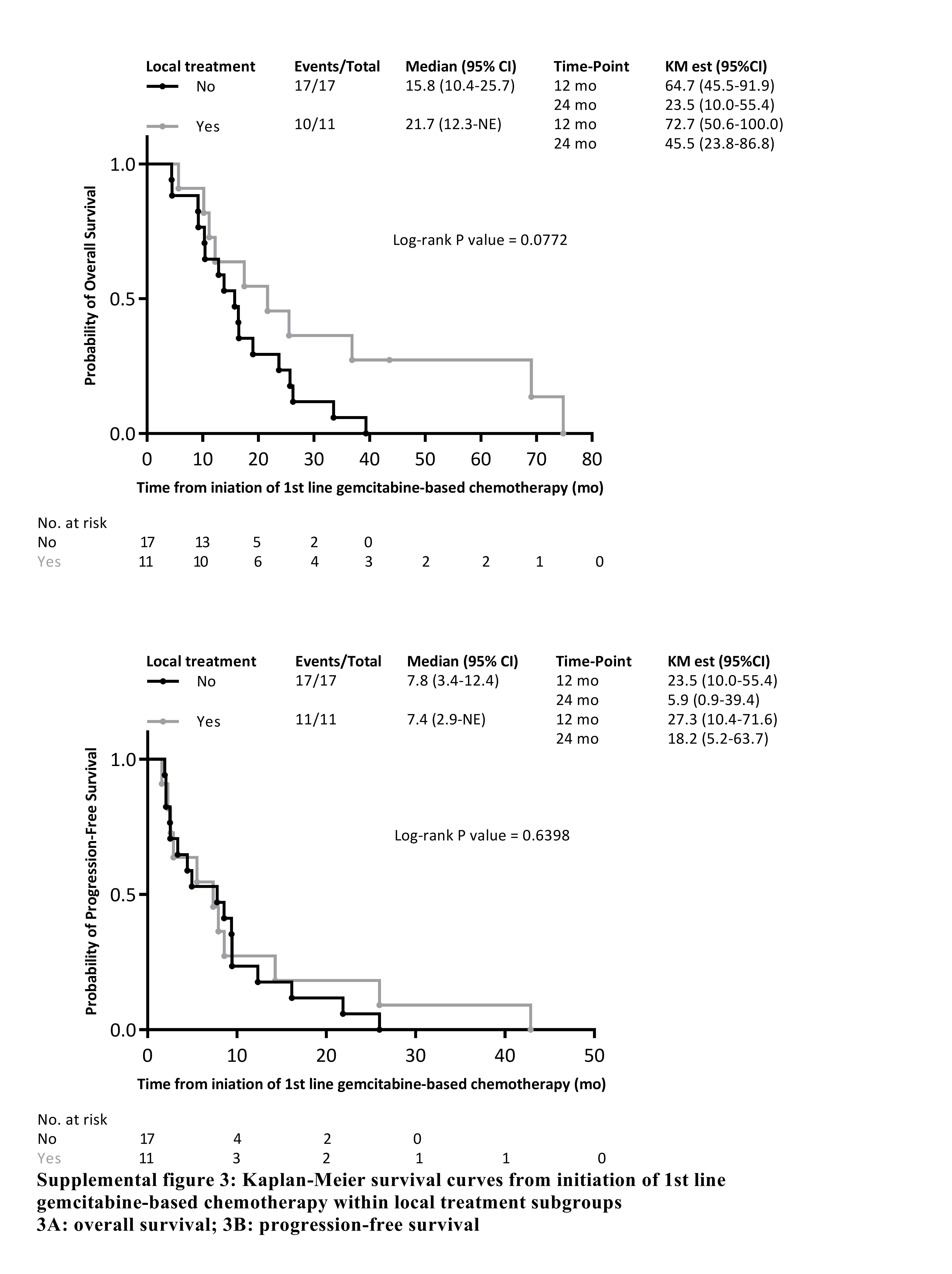

Supplement: Supplementary file 4 [file Image_3.jpeg]
